# 

**DOI:** 10.1192/bjb.2022.98

**Published:** 2023-12

**Authors:** Tom Harrison

**Affiliations:** Honorary Researcher at the History of Medicine Unit, University of Birmingham, UK. Email: tomharrisonjw@gmail.com

**Figure:**
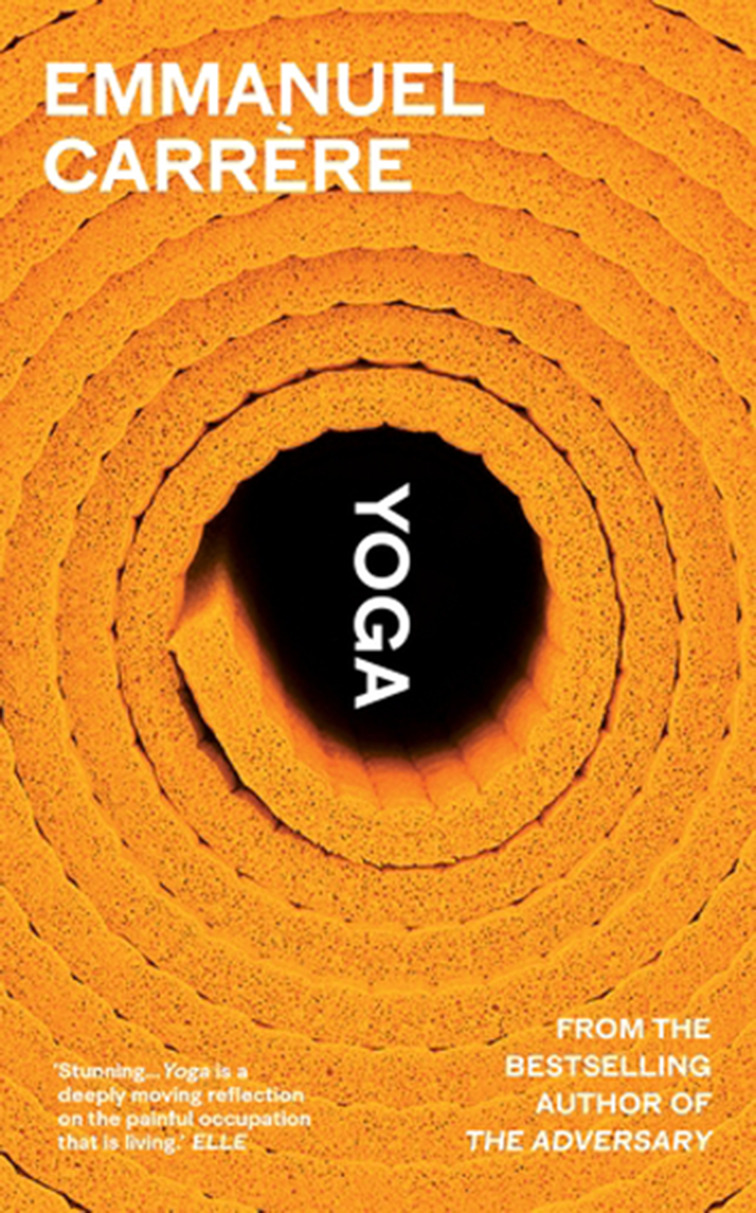

Distinguished author Emmanuel Carrère wrote *Yoga*, an account of his bipolar disorder and his preoccupation with meditation, when he was ‘almost sixty’ years old. One has to be a little careful in reading this book. In his own words some of it is fictional and his ex-wife alleges that it ‘is completely false’.

Attending a 10-day meditation retreat, he describes at length his innermost thoughts. However, there is a sense of his not ‘being in the moment’. He plans to write his experiences up as a ‘subtle little book on yoga’ and is constantly observing and recording, defining meditation in 25 different ways.

On 7 January 2015, Bernard Maris was shot dead at the headquarters of the satirical magazine *Charlie Hebdo* and Carrère returns to support the man's partner. Soon his thoughts come ‘thick and fast, twist like flames, burn themselves out and ignite all over again’. Unaware that his mood swings were unusual, he had previously seen himself as ‘wafted gently by the current between the two poles’, the *yin* and *yang* of affect. Now he remains in his shapeless corduroy trousers, an old sweater full of holes and trainers without laces, oscillating between bed and smoking in a dazed stupor in the local café. Admitted to hospital for 4 months, he is uncertain whether the ECT was helpful. His memory loss concerns him, as he cannot recall the events of his stay on the secure ward.

Writing about one's journey into mental illness one has to steer between the Charybdis of self-absorption and the Scylla of objective rationality in order to inform and maintain the reader's attention. All too often the internal chaos, despair and horror becomes all absorbing and is only interesting to oneself, rather like telling others your dreams. Carrère introduces an outside perspective when a journalist describes how he looked ‘forlorn and abandoned’, but this book still hovers on the fringe. There is a vivid passage describing the horrors of depression but, overall, it illustrates his personality more than his illness.

Psychiatrists should be aware of the subjective experience of mental disorder, and personal accounts are essential reading. However, I have already suggested that, without editing, such stories can become tedious and self-absorbed. Maybe we should see this book as being in the tradition of Evelyn Waugh's *The Ordeal of Gilbert Pinfold*, in which the author dresses up his own experience in fictional terms.

